# Association of Individual-Level Factors With Visual Outcomes in Optic Neuritis: Secondary Analysis of a Randomized Clinical Trial

**DOI:** 10.1001/jamanetworkopen.2020.4339

**Published:** 2020-05-07

**Authors:** Lindsey B. De Lott, James F. Burke, Chris A. Andrews, Fiona Costello, Wayne T. Cornblath, Jonathan D. Trobe, Paul P. Lee, Kevin A. Kerber

**Affiliations:** 1 Department of Ophthalmology and Visual Sciences, University of Michigan, Ann Arbor; 2 Department of Neurology, University of Michigan, Ann Arbor; 3 Section of Ophthalmology, Department of Clinical Neurosciences and Surgery, University of Calgary, Calgary, Alberta, Canada

## Abstract

**Importance:**

Using corticosteroids to treat acute demyelinating optic neuritis has been identified as an area for shared decision-making. However, no analysis exists to support personalized shared decision-making that considers long- and short-term treatment benefits.

**Objective:**

To develop models of individual-level visual outcomes for patients with optic neuritis.

**Design, Setting, and Participants:**

This secondary analysis of the Optic Neuritis Treatment Trial (ONTT), a randomized clinical trial, was performed at 14 academic eye centers and 1 large community eye center. Adults aged 18 to 46 years with incident acute unilateral optic neuritis within 8 days of vision loss onset were included. Data were collected from July 1988 to June 1991, downloaded on October 15, 2018, and analyzed from January 24, 2019, to February 20, 2020, using multivariable linear regression modeling.

**Exposures:**

Intravenous corticosteroids vs placebo.

**Main Outcomes and Measures:**

Visual acuity (VA) at 1 year. Secondary outcomes were 1-year contrast sensitivity (CS) and VA and CS at 15 and 30 days. Independent variables included age, sex, race, multiple sclerosis status, optic neuritis episodes in the fellow eye, vision symptoms (days), pain, optic disc swelling, viral illness, treatment group, and baseline VA or CS.

**Results:**

Of the 455 participants, median age was 31.8 (interquartile range [IQR], 26.3-37.0) years; 350 (76.9%) were women; and 388 (85.3%) were white. For 410 participants (90.1%) with 1-year outcomes, median VA improved from 20/66 (IQR, 20/28-20/630) at enrollment to 20/17 (IQR, 20/14-20/21) at 1 year. Baseline VA was the primary variable associated with 1-year VA (regression coefficient, 0.056 [95% CI, 0.008-0.103]; *P* = .02) if baseline VA was better than count fingers (CF). At 15 days, baseline VA and treatment status were associated with VA in those participants with baseline VA better than CF (regression coefficient, 0.305 [95% CI, 0.231-0.380]; *F* = 9.42; *P* < .001). However, the difference of medians (20/18 [95% CI, 20/17-20/19] with intravenous corticosteroids vs 20/23 [95% CI, 20/21-20/26] with placebo) was small for the median VA (20/66) in the trial. Treatment was not associated with 15-day or 1-year VA in participants with baseline VA of CF or worse.

**Conclusions and Relevance:**

In this study, long-term VA was associated with severity of baseline vision loss. Early benefits with intravenous corticosteroid treatment were limited to participants with baseline VA better than CF. However, the early, temporary benefit of intravenous corticosteroids is of questionable clinical significance and should be weighed against potential harms.

## Key Points

**Question:**

What individual-level factors are associated with visual acuity in patients with optic neuritis?

**Findings:**

In this secondary analysis of clinical trial data of 455 patients with optic neuritis, baseline visual acuity was associated with 1-year visual acuity, and baseline visual acuity and treatment status were associated with visual acuity at 15 days. However, for the median baseline visual acuity of 20/66, the difference of medians in visual acuity at 15 days was small with intravenous corticosteroids (20/18) compared with placebo (20/23).

**Meaning:**

In this study, the primary factor associated with long-term visual acuity was severity of baseline vision impairment, and the temporary early benefit of intravenous corticosteroids was of questionable clinical importance.

## Teaser

This secondary analysis of a randomized clinical trial develops models of individual-level visual outcomes for patients with optic neuritis.

## Introduction

The Optic Neuritis Treatment Trial (ONTT) was a landmark randomized clinical trial showing that treatment with corticosteroids does not improve long-term visual outcomes for patients with acute demyelinating typical optic neuritis but may hasten visual recovery in the first 2 weeks.^1,2,3^ Given the lack of a robust treatment effect with corticosteroids, the ONTT Study Group and clinical practice guidelines called for shared decision-making between patients and physicians when deciding whether to use corticosteroids for acute neuritis.^4^ Since the publication of the ONTT findings, the diagnosis of typical optic neuritis remains unchanged, and no additional short-term treatments have emerged. Therefore, the ONTT provides the only actionable data to date for counseling patients on the benefits of corticosteroids.

Despite the lack of corticosteroid effect on long-term outcomes, more than 90% of neurologists report treating patients with optic neuritis with corticosteroids.^5^ Patient preference was the least cited reason for treatment, suggesting shared decision-making is not occurring.^5,6^ One barrier to the application of shared decision-making is that clinicians do not have information that considers individual characteristics, such as severity of the visual deficit, and how those individual characteristics might influence long-term recovery or likelihood to experience a hastened recovery.^5^ Personalizing clinical trial information is a strategy used to address this barrier and ultimately promote informed, shared decision-making.^7^ Currently, we know of no studies that allow clinicians to provide personalized information regarding visual recovery and short-term benefit with corticosteroids to patients with optic neuritis.

The purpose of our study was to perform a multivariable, risk-stratified analysis of ONTT data to assess visual function over time and at 1 year at the individual level. We hypothesized that several baseline factors would be associated with vision outcomes over time. Ultimately, this information could support shared decision-making.

## Methods

### Standard Protocol Approvals, Registrations, and Patient Consent

Because ONTT data are deidentified and publicly available,^8^ our study was determined to have “not regulated” status by the University of Michigan’s institutional review board in accordance with federal regulations regarding human subjects research. A waiver of consent was not required. The reporting of this study conforms to the Strengthening the Reporting of Observational Studies in Epidemiology (STROBE) reporting guideline.^9^

### Study Population

We conducted a secondary analysis of data from the ONTT (data downloaded on October 15, 2018), which enrolled patients from July 1988 to June 1991 and then continued to follow up patients for 15 years. Analyses were performed from January 24, 2019, to February 20, 2020. The ONTT is the most comprehensive data source for optic neuritis because of the large number of patients, wide range of clinically important patient variables collected, systematic measurement of baseline variables, and low attrition rates.

The ONTT enrolled 457 people at 14 academic eye centers and 1 large community eye center. Participants were aged 18 to 46 years with incident acute unilateral optic neuritis within 8 days of onset of vision loss as determined by the neuro-ophthalmologist site investigator using standard criteria.^1,6^ Study visits after enrollment occurred at 4, 15, and 30 days and 7, 13, 19, 26, and 52 weeks. Yearly visits occurred thereafter.^6^

### Independent Variables

Independent variables in the model were age, sex, race (white vs nonwhite), history of multiple sclerosis (MS; none, possible, probable, or definite, based on Poser criteria^10^), number of optic neuritis episodes in the fellow eye, days of vision loss, pain, optic disc swelling, viral illness within 1 month, and baseline VA (for the VA model only) or baseline contrast sensitivity (CS; for the CS model only). Given the limited magnetic resonance imaging data, it was not included as an independent variable.

Visual acuity was measured in the ONTT using retroilluminated Early Treatment of Diabetic Retinopathy Study (ETDRS) charts and converted to the logarithm of the minimal angle of resolution (logMAR) for analysis purposes. A standard logMAR equivalent for patients with VA of count fingers (CF), hand motion, light perception, and no light perception does not exist.^11,12^ The original ONTT analysis assigned a logMAR VA of 1.7 for all participants with VA of CF or less. For analyses requiring numeric values, we used 1.85 for CF, 2.30 for hand motion, 2.75 for light perception, and 3.20 for no light perception. These measurements are similar to previously used values and permitted easy display of the data.^11^ Conclusions from models were insensitive to our choices. For reporting results, all logMAR values were converted back to Snellen equivalents. Contrast sensitivity was measured in the ONTT using Pelli-Robson charts, which use letters that subtend 2.8° at 1 m. Letters are arranged in groups of 3 with 16 steps of decreasing contrast of 0.15 log units, with each step relative to the chart background (range, 0-16 steps).

### Outcome Variables

Our primary outcome was VA at 1 year. Secondary outcomes were CS at 1 year and VA and CS at days 15 and 30.

### Statistical Analysis and Prediction Models

Demographic and baseline clinical characteristics of participants who did not complete the 1-year visit were compared with those who did using 2-tailed *t* tests for continuous data and Fisher exact test for categorical data; 2-sided *P* < .05 indicated significance. Multiple linear regression models were built to estimate VA and CS at 15 and 30 days and 1 year using a prespecified plan to evaluate model assumptions. The models were built using all available ONTT data at each point. Model discrimination, which is the variability of the outcome measure explained by the independent variables, was estimated using adjusted R^2^ values. Calibration, which is an assessment of how closely estimated values represent true values, was assessed using numeric and graphical analysis.

For the VA models, first we used ordinary least squares regression; however, regression diagnostics revealed multiple violations of ordinary least squares regression assumptions, including residuals with heteroscedastic variance; nonlinearity of the association between the independent and outcome variables; and nonnormal, skewed residuals. Robust regression (M-estimation) and generalized additive models were considered, but both still violated assumptions when using all observations. After exploring our data, we determined that patients with very low baseline acuity were driving model assumption violations, and so we stratified the data (VA better than CF vs CF or worse) for analysis. Analysis within these subsets did not demonstrate the noted violations. Therefore, separate subset linear regression models were used to estimate VA outcome in those participants with a baseline VA better than CF and the estimated VA outcome in those participants with a baseline VA of CF or worse. Likewise, separate subset linear regression models were used to estimate CS outcome in those participants with a baseline CS better than 0 and those with baseline CS of 0. We performed sensitivity analyses using dichotomous outcomes of 20/20 or better and 20/40 or better for VA and CS of 12 or better. Results were similar to the analyses presented.

Heterogeneity of treatment effect was investigated for both the VA and CS models by including interactions between baseline vision and treatment group. The 95% predictions intervals for individuals were made based on quantile regressions of the response variable on baseline VA or CS. Statistical analyses were performed using R, version 3.6.0 (R Project for Statistical Computing).

## Results

Of the 455 participants (457 randomized; 2 excluded for compressive optic neuropathy), the median age was 31.8 (interquartile range [IQR], 26.3-37.0) years; 350 (76.9%) were women, 105 (23.1%) were men, and 388 (85.3%) were white (Table 1). Median VA at baseline was 20/66 (IQR, 20/28-20/630). Median CS at baseline was 9 (IQR, 4-12). Median time from onset of vision loss to treatment was 5 (IQR, 4-6) days.

**Table 1.** Baseline Characteristics of the Study Population for All Enrolled Participants and Those Completing the 1-Year Visit

Variable	Patient group^a^	
	All enrolled (n = 455)^b^	Completed the 1-y evaluation (n = 410)
Age, median (IQR), y	31.8 (26.3-37.0)	31.9 (26.4-37.5)
Female	350 (76.9)	319 (77.8)
Race		
White	388 (85.3)	357 (87.1)
Black	58 (12.7)	46 (11.2)
Other	9 (2.0)	7 (1.7)
Pain	417 (91.6)	374 (91.2)
Optic disc edema	161 (35.4)	142 (34.6)
≥1 prior optic neuritis episode in fellow eye	32 (7.0)	29 (7.1)
Duration of symptoms, median (IQR), d	5 (4-6)	5 (4-6)
Preceding viral illness	119 (26.2)	108 (26.3)
MS status^c^		
None	314 (69.2)	285 (69.5)
Possible	75 (16.5)	68 (16.6)
Probable	29 (6.4)	22 (5.4)
Confirmed	36 (7.9)	35 (8.5)
Treatment assignment		
Placebo	148 (32.5)	133 (32.4)
Oral prednisone	156 (34.3)	140 (34.1)
Intravenous methylprednisolone	151 (33.2)	137 (33.4)
Baseline Snellen VA		
Median (IQR)	20/66 (20/28 to 20/632)	20/63 (20/28 to 20/604)
CF or worse	73 (16.0)	63 (15.4)
20/20 or better	50 (11.0)	46 (11.2)
Baseline CS		
Median (IQR)	9 (4-12)	10 (4-12)
<12	285 (62.6)	255 (62.2)

Abbreviations: CF, count fingers; CS, contrast sensitivity; IQR, interquartile range; MS, multiple sclerosis; VA, visual acuity.

^a^ Unless otherwise indicated, data are expressed as number (percentage) of patients.

^b^ Two participants from the original cohort were excluded for compressive optic neuropathy.

^c^ One participant was missing multiple sclerosis data at baseline (n = 454). Multiple sclerosis was classified using Poser criteria.

Participant retention was 442 (97.1%) at 15 days, 433 (95.2%) at 30 days, and 410 (90.1%) at 1 year. Median VA was 20/20 (IQR, 20/16-20/38) at 15 days, 20/18 (IQR, 20/16-20/25) at 30 days, and 20/17 (IQR, 20/14-20/21) at 1 year (211 of 270 [78.1%] with 20/20 or better and 256 of 270 [94.8%] with 20/40 or better). Visual acuity was worse than 20/20 in 220 of 442 participants (49.8%) at 15 days, 173 of 433 (40.0%) at 30 days, and 113 of 410 (27.6%) at 1 year. After excluding 166 participants who received oral prednisone, 59 of 270 (21.9%) had a VA worse than 20/20 at 1 year and 14 of 270 (5.2%) had a VA worse than 20/40 at 1 year. Median CS was 13 (IQR, 10-14) at 15 days, 14 (IQR, 12-15) at 30 days, and 14 (IQR, 14-15) at 1 year. Contrast sensitivity was 12 or worse in 212 of 442 participants (48.0%) at 15 days, 159 of 433 (36.7%) at 30 days, and 68 of 410 (16.6%) at 1 year. Compared with those who attended the 1-year follow-up visit, participants who missed the 1-year follow-up visit were more likely to be nonwhite (14 of 45 [31.1%] vs 53 of 410 [12.9%]; *P* = .003), to have probable MS at baseline (7 of 44 [15.9%] vs 22 of 410 [5.4%]) rather than definite (1 of 44 [2.3%] vs 35 of 410 [8.5%]; *P* = .045), and to be younger (median age, 30.2 [IQR, 24.6-33.7] vs 31.9 [IQR, 26.4-37.5] years; *P* = .02). Plots of VA and CS at 1 year vs baseline are presented in the Figure and demonstrate the overall recovery of vision for most participants, but with a greater range of observed outcomes for those with VA of light perception or no light perception at baseline.

**Figure.**
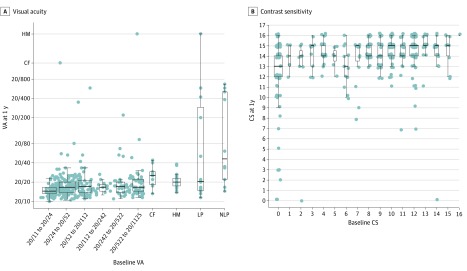
Box Plots of Visual Function Visual acuity (VA) at 1 year vs baseline and contrast sensitivity (CS) at 1 year vs baseline are shown. Grey points represent subjects. Box plots are created by binning the logarithm of the minimal angle of resolution. Widths of boxes are proportional to the square root of the number of participants in the bin. CF indicates count fingers; HM, hand motion; LP, light perception; and NLP, no light perception.

In multiple linear regression models of VA at 1 year, only baseline VA was associated with long-term VA (coefficient, 0.056 [95% CI, 0.008-0.103]; *P* = .02) for participants with a baseline VA better than CF (Table 2) (n = 347). The 1-year model had poor discrimination, explaining only 0.6% of the variance in 1-year VA outcome, because the outcome had low variability across participants with VA better than CF. For participants with a baseline VA of CF or worse, baseline VA was also associated with VA at 1 year (*F* = 5.57; *P* = .002) (n = 63) (Table 3). Among participants with baseline VA worse than CF and possible MS, VA at 1 year was significantly worse compared with those without MS (coefficient, 0.546 [95% CI, 0.098-1.000]; *P* = .02). The interaction term of baseline vision and treatment group was not statistically significant, indicating lack of evidence of a heterogeneity of treatment effect.

**Table 2.** Multivariable Linear Regression Models of Visual Outcomes at 15 and 30 Days and 1 Year in Participants With Baseline VA Better Than CF and CS Better Than 0 Pelli-Robson Steps

Variable	Study visit, VA						Study visit, CS					
	15 d (n = 370)		30 d (n = 367)		1 y (n = 347)^a^		15 d (n = 358)		30 d (n = 355)		1 y (n = 335)	
	Regression coefficient (95% CI)	*P* value	Regression coefficient (95% CI)	*P* value	Regression coefficient (95% CI)	*P* value	Regression coefficient (95% CI)	*P* value	Regression coefficient (95% CI)	*P* value	Regression coefficient (95% CI)	*P* value
Constant	0.235 (−0.065 to 0.534)	.12	−0.024 (−0.233 to 0.184)	.82	−0.111 (−0.306 to 0.083)	.26	7.868 (5.629 to 10.108)	<.001	11.063 (9.391 to 12.735)	<.001	13.754 (12.190 to 15.318)	<.001
Baseline logMAR VA (per unit) or CS (per step)	0.305 (0.231 to 0.380)	<.001	0.127 (0.075 to 0.178)	<.001	0.056 (0.008 to 0.103)	.02	0.291 (0.205 to 0.378)	<.001	0.203 (0.139 to 0.267)	<.001	0.084 (0.024 to 0.143)	.006
Treatment comparison between groups^b^	NA	<.001	NA	.15	NA	.20	NA	<.001	NA	.10	NA	.48
Oral prednisone vs placebo	−0.105 (−0.205 to −0.005)	.04	0.015 (−0.055 to 0.085)	.67	0.028 (−0.037 to 0.093)	.40	1.128 (0.438 to 1.819)	.001	0.169 (−0.347 to 0.685)	.52	−0.055 (−0.538 to 0.4282)	.82
Intravenous methylprednisolone vs placebo	−0.226 (−0.328 to −0.123)	<.001	−0.051 (−0.122 to 0.020)	.16	−0.031 (−0.097 to 0.035)	.35	2.058 (1.346 to 2.770)	<.001	0.567 (0.037 to 1.097)	.04	0.225 (−0.268 to 0.718)	.37
Age per year	0.001 (−0.005 to 0.007)	.81	0.004 (−0.001 to 0.008)	.10	0.002 (−0.002 to 0.006)	.38	−0.010 (−0.052 to 0.033)	.66	−0.031 (−0.0624 to 0.001)	.06	−0.023 (−0.052 to 0.007)	.13
Female	0.080 (−0.018 to 0.179)	.11	0.015 (−0.054 to 0.084)	.67	−0.002 (−0.067 to 0.064)	.96	−1.083 (−1.764 to −0.403)	.002	−0.598 (−1.105 to −0.091)	.02	−0.447 (−0.929 to 0.035)	.07
Nonwhite	0.017 (−0.113 to 0.147)	.80	−0.023 (−0.111 to 0.066)	.62	0.026 (−0.059 to 0.110)	.55	−0.573 (−1.467 to 0.322)	.21	−0.105 (−0.756 to 0.547)	.75	−0.072 (−0.694 to 0.550)	.82
MS status, comparison between groups	NA	.54	NA	.31	NA	.37	NA	.82	NA	.11	NA	.68
Possible	−0.033 (−0.147 to 0.081)	.57	−0.054 (−0.133 to 0.025)	.18	0.061 (−0.013 to 0.134)	.10	−0.036 (−0.824 to 0.752)	.93	0.262 (−0.322 to 0.845)	.38	−0.254 (−0.794 to 0.286)	.36
Probable	0.108 (−0.078 to 0.294)	.25	0.067 (−0.065 to 0.200)	.32	0.052 (−0.086 to 0.189)	.46	−0.604 (−1.931 to 0.724)	.37	−0.470 (−1.483 to 0.544)	.36	−0.323 (−1.393 to 0.747)	.55
Definite	0.045 (−0.114 to 0.204)	.58	−0.039 (−0.150 to 0.073)	.50	−0.005 (−0.107 to 0.096)	.92	0.128 (−0.964 to 1.220)	.82	0.872 (0.048 to 1.696)	.04	0.170 (−0.578 to 0.912)	.66
Pain	−0.042 (−0.185 to 0.101)	.57	0.003 (−0.097 to 0.103)	.95	0.010 (−0.083 to 0.102)	.84	1.033 (−0.097 to 2.163)	.07	0.265 (−0.577 to 1.106)	.54	−0.196 (−0.981 to 0.589)	.62
Optic disc edema	−0.011 (−0.098 to 0.076)	.81	0.010 (−0.051 to 0.070)	.76	0.009 (−0.048 to 0.065)	.76	0.215 (0.035 to 0.395)	.02	0.190 (0.056 to 0.325)	.006	0.105 (−0.020 to 0.230)	.10
Prior optic neuritis	−0.156 (−0.320 to 0.009)	.06	−0.061 (−0.175 to 0.054)	.30	−0.021 (−0.127 to 0.086)	.71	0.124 (−0.887 to 1.135)	.81	0.157 (−0.596 to 0.909)	.68	0.041 (−0.660 to 0.742)	.91
Duration of symptoms (per day)	−0.031 (−0.057 to −0.005)	.02	−0.023 (−0.041 to −0.004)	.02	−0.008 (−0.025 to 0.009)	.36	0.091 (−0.511 to 0.693)	.77	0.002 (−0.445 to 0.450)	.99	−0.158 (−0.573 to 0.257)	.45
Preceding viral illness	0.018 (−0.075 to 0.110)	.71	−0.017 (−0.0807 to 0.047)	.61	−0.033 (−0.093 to 0.026)	.27	0.176 (−0.455 to 0.807)	.58	0.138 (−0.330 to 0.607)	.56	0.132 (−0.304 to 0.569)	.55
Adjusted *R*^2^ value, %	18.3	NA	7.3	NA	0.6	NA	18.0	NA	12.7	NA	2.3	NA

Abbreviations: CF, count fingers; CS, contrast sensitivity; logMAR, logarithm of the minimal angle of resolution; MS, multiple sclerosis; NA, not applicable; VA, visual acuity.

^a^ Formula for expected VA in logMAR at 1 year: expected VA = −0.111 + 0.056 VA_0 + 0.028 oral prednisone −0.031 intravenous methylprednisolone +0.002 age −0.002 female +0.026 nonwhite +0.061 possible MS + 0.052 probable MS − 0.005 definite MS +0.010 pain +0.009 optic disc edema −0.021 prior optic neuritis −0.008 symptom days −0.033 viral illness.

^b^ Calculated as analysis of variance *F* test of overall factor effect.

**Table 3.** Multivariable Linear Regression Models of Visual Outcomes at 15 and 30 Days and 1 Year in Participants With Baseline VA of CF or Worse and With CS of 0 Pelli-Robson Steps

Variable	Study visit, VA						Study visit, CS steps					
	15-d (n = 72)		30-d (n = 66)		1-y (n = 63)		15 d (n = 84)		30 d (n = 78)		1 y (n = 75)	
	Regression coefficient (95% CI)	*P* value	Regression coefficient (95% CI)	*P* value	Regression coefficient (95% CI)	*P* value	Regression coefficient (95% CI)	*P* value	Regression coefficient (95% CI)	*P* value	Regression coefficient (95% CI)	*P* value
Constant	0.498 (−0.952 to 1.947)	.49	−0.864 (−2.044 to 0.317)	.15	−0.260 (−1.202 to 0.682)	.58	5.523 (−2.077 to 13.122)	.15	14.905 (7.832 to 21.978)	<.001	17.431 (11.569 to 23.293)	<.001
Baseline VA comparison between groups	NA	.01	NA	.06	NA	.002	NA	NA	NA	NA	NA	NA
HM vs CF	0.189 (−0.407 to 0.784)	.53	0.173 (−0.313 to 0.660)	.48	−0.208 (−0.602 to 0.187)	.30	NA	NA	NA	NA	NA	NA
LP vs CF	0.361 (−0.324 to 1.046)	.30	0.206 (−0.369 to 0.782)	.48	0.269 (−0.190 to 0.729)	.25	NA	NA	NA	NA	NA	NA
NLP vs CF	1.181 (0.469 to 1.893)	.002	0.816 (0.220 to 1.412)	.008	0.629 (0.138 to 1.120)	.01	NA	NA	NA	NA	NA	NA
Treatment comparison between groups^a^	NA	.20	NA	.47	NA	.38	NA	.16	NA	.52	NA	.61
Oral prednisone vs placebo	−0.390 (−0.998 to 0.217)	.20	−0.323 (−0.845 to 0.199)	.22	0.179 (−0.209 to 0.567)	.36	−0.364 (−3.560 to 2.833)	.82	−1.244 (−4.214 to 1.726)	.41	−1.096 (−3.538 to 1.345)	.37
Intravenous methylprednisolone vs placebo	−0.495 (−1.042 to 0.053)	.08	−0.186 (−0.638 to 0.266)	.41	0.246 (−0.108 to 0.600)	.17	2.088 (−0.737 to 4.913)	.15	0.221 (−2.388 to 2.839)	.89	−0.925 (−3.093 to 1.243)	.40
Age per year	0.015 (−0.025 to 0.054)	.47	0.010 (−0.022 to 0.043)	.53	0.001 (−0.025 to 0.027)	.93	−0.032 (−0.221 to 0.157)	.74	−0.058 (−0.234 to 0.118)	.51	−0.069 (−0.212 to 0.075)	.34
Female	0.381 (−0.174 to 0.935)	.17	−0.022 (−0.479 to 0.434)	.92	0.205 (−0.163 to 0.573)	.27	−2.589 (−5.547 to 0.369)	.09	−0.549 (−3.324 to 2.226)	.69	−0.872 (−3.203 to 1.459)	.46
Nonwhite	−0.235 (−0.735 to 0.265)	.35	0.182 (−0.248 to 0.612)	.40	−0.045 (−0.397 to 0.307)	.80	0.036 (−2.710 to 2.782)	.98	−1.760 (−4.401 to 0.881)	.19	−0.260 (−2.544 to 2.023)	.82
MS status comparison between groups	NA	.46	NA	.22	NA	.04	NA	.56	NA	.24	NA	.26
Possible vs no	0.022 (−0.656 to 0.699)	.95	0.302 (−0.242 to 0.845)	.27	0.549 (0.098 to 1.000)	.02	0.459 (−2.924 to 3.841)	.79	−2.791 (−5.888 to 0.306)	.08	−2.531 (−5.183 to 0.121)	.06
Probable vs no	−0.566 (−1.303 to 0.170)	.13	−0.434 (−0.991 to 0.123)	.12	−0.236 (−0.678 to 0.206)	.29	0.778 (−2.996 to 4.551)	.68	0.964 (−2.307 to 4.235)	.56	0.433 (−2.253 to 3.118)	.75
Definite vs no	−0.200 (−1.032 to 0.631)	.63	−0.142 (−0.871 to 0.588)	.70	0.199 (−0.321 to 0.719)	.45	3.103 (−1.265 to 7.470)	.16	0.830 (−3.438 to 5.099)	.70	0.041 (−3.174 to 3.256)	.98
Pain	0.937 (−0.191 to 2.065)	.10	0.006 (−0.874 to 0.885)	.99	−0.044 (−0.746 to 0.657)	.90	−3.881 (−9.570 to 1.808)	.18	−0.738 (−5.810 to 4.334)	.77	−0.251 (−4.420 to 3.918)	.91
Optic disc edema	−0.019 (−0.173 to 0.135)	.80	0.087 (−0.054 to 0.228)	.22	0.032 (−0.072 to 0.136)	.54	0.294 (−0.465 to 1.052)	.44	−0.092 (−0.857 to 0.672)	.81	−0.213 (−0.811 to 0.386)	.48
Prior optic neuritis	−0.071 (−1.109 to 0.968)	.89	0.326 (−0.488 to 1.140)	.43	−0.046 (−0.695 to 0.602)	.89	1.117 (−3.577 to 5.811)	.64	−1.149 (−5.338 to 3.040)	.59	−0.613 (−4.065 to 2.839)	.72
Duration of symptoms per day	−0.109 (−0.631 to 0.414)	.68	−0.111 (−0.532 to 0.310)	.60	−0.249 (−0.611 to 0.112)	.17	−0.379 (−3.070 to 2.312)	.78	0.787 (−1.702 to 3.275)	.53	0.930 (−1.216 to 3.076)	.39
Preceding viral illness	0.612 (0.068 to 1.156)	.03	0.483 (0.049 to 0.918)	.03	0.216 (−0.148 to 0.580)	.24	−1.589 (−4.529 to 1.351)	.29	−2.566 (−5.248 to 0.116)	.06	−2.090 (−4.380 to 0.200)	.07
Adjusted* R*^2^ value, %	11.7	NA	10.3	NA	18.6	NA	0.03	NA	3.1	NA	0.1	NA

Abbreviations: CF, count fingers; CS, contrast sensitivity; HM, hand motion; LP, light perception; MS, multiple sclerosis; NA, not applicable; NLP, no light perception; VA, visual acuity.

^a^ Calculated as analysis of variance *F* test of overall factor effect.

The model of VA at the 15-day visit for participants with baseline VA better than CF found that baseline VA (coefficient, 0.305 [95% CI, 0.231-0.380]; *P* < .001), treatment with intravenous corticosteroids (−0.226 [95% CI, −0.328 to −0.123]; *P* < .001) and oral corticosteroids (−0.105 [95% CI, −0.205 to −0.005]; *P* = .04) vs placebo, and duration of vision loss (in days) before enrollment (−0.031 [95% CI, −0.057 to −0.005]; *P* = .02) were associated with VA in participants with a baseline VA better than CF (Table 2). However, at the 30-day visit, no association with VA was found for treatment with intravenous (−0.051 [95% CI, −0.122 to 0.020]; *P* = .16) or oral (0.015 [95% CI, −0.055 to 0.085]; *P* = .67) corticosteroids. The models of VA at the 15- and 30-day visits for participants with baseline VA of CF or worse found that only baseline VA (F = 4.12; *P* = .01) and preceding viral illness was associated with VA at 15 days (0.612 [95% CI, 0.068-1.156]; *P* = .023) and 30 days (0.483 [95% CI, 0.049-0.918]; *P* = .03) (Table 3). Results from multiple linear regression models of CS at 15 and 30 days and 1 year are found in Tables 2 and 3.

For the median baseline VA of 20/66, the median VA at 15 days was 20/18 (95% CI, 20/17-20/19) with intravenous corticosteroid use vs 20/23 (95% CI, 20/21-20/26) with placebo use, but no difference was detected at 30 days (20/17 [95% CI, 20/16-20/19] for intravenous corticosteroid use vs 20/19 [95% CI, 20/18-20/20] for placebo use) or 1 year (20/16 [95% CI, 20/15-20/17] for intravenous corticosteroid and placebo). Table 4 and eTable in the Supplement (in meters [Snellen], Glasgow Acuity Cards notation, and logMAR) estimate VA at 15 and 30 days and 1 year compared with the actual 1-year outcomes. Vision categorization was based on the World Health Organization definitions of vision impairment.^13^ Model estimates were well calibrated to observed outcomes.

**Table 4.** Estimated VA at 15 and 30 Days and 1 Year and Observed VA at 1 Year Based on Baseline VA and Treatment

Baseline VA by treatment^a^	Estimated VA (95% estimation interval)			Observed 1-y VA			
	15 d	30 d	1 y	No. of participants	Tukey summary, median (IQR) [range]	Worse than 20/20, No. (%) [95% CI]^b^	Worse than 20/40, No. (%) [95% CI]^b^
Better than 20/30							
Placebo	20/20 (20/14-20/66)	20/17 (20/13-20/29)	20/15 (20/11-20/24)	47	20/15 (20/13 to 20/17) [20/11 to 20/32]	7 (14.9) [7.4-27.7	0 (0) (0-7.6)
Intravenous corticosteroid	20/15 (20/12-20/25)	20/16 (20/11-20/25)	20/15 (20/11-20/22)	35	20/15 (20/14-20/17) [20/12-20/24]	2 (5.7) [1.6-18.6]	0 (0) [0-9.9]
20/30-20/60							
Placebo	20/22 (20/14-20/126)	20/19 (20/13-20/43)	20/16 (20/11-20/31)	26	20/18 (20/15-20/20) [20/12-20/28]	6 (23.1) [11.0-42.0]	0 (0) [0-12.9]
Intravenous corticosteroid	20/17 (20/12-20/48)	20/17 (20/11-20/37)	20/16 (20/11-20/28)	29	20/17 (20/13-20/20) [20/10-20/348]	6 (20.7) [9.8-38.4]	1 (3.4) [0.6-17.2]
20/70-20/160							
Placebo	20/25 (20/15-20/263)	20/20 (20/14-20/68)	20/16 (20/12-20/41)	15	20/16 (20/14-20/20) [20/12-20/577]	4 (26.7) [10.9-51.9]	2 (13.3) [3.7-37.9]
Intravenous corticosteroid	20/19 (20/13-20/100)	20/18 (20/12-20/58)	20/16 (20/11-20/37)	21	20/16 (20/13-20/18) [20/11-20/33]	4 (19.0) [7.7-40.0]	0 (0) [0-15.5]
20/200-20/400							
Placebo	20/29 (20/15-20/582)	20/21 (20/14-20/110)	20/17 (20/12-20/56)	10	20/18 (20/16-20/20) [20/14-20/219]	2 (20.0) [5.7-51.0]	1 (10.0) [1.8-40.4]
Intravenous corticosteroid	20/22 (20/13-20/221)	20/19 (20/12-20/94)	20/16 (20/11-20/50)	14	20/16 (20/13-20/17) [20/11-20/66]	2 (14.3) [4.0-39.9]	2 (14.3) [4.0-39.9]
20/500-20/1000							
Placebo	20/32 (20/16-20/1181)	20/23 (20/14-20/171)	20/17 (20/12-20/73)	15	20/17 (20/14-20/19) [20/12-20/30]	3 (20.0) [7.0-45.2]	0 (0) [0-20.4]
Intravenous corticosteroid	20/25 (20/14-20/449)	20/21 (20/12-20/146)	20/17 (20/12-20/65)	17	20/17 (20/14-20/18) [20/12-20/91]	2 (11.8) [3.3-34.3]	1 (5.9) [1.0-27.0]
CF and HM							
Placebo	20/53 (20/23-HM)	20/25 (20/16-20/680)	20/22 (20/14-20/39)	13	20/22 (20/17-20/25) [20/13-20/44]	7 (53.8) [29.1-76.8]	1 (7.7) [1.4-33.3]
Intravenous corticosteroid	20/24 (20/15-20/733)	20/21 (20/13-20/470)	20/21 (20/17-20/39)	12	20/21 (20/19-20/22) [20/16-20/40]	6 (50.0) [25.4-74.6]	0 (0) [0-24.2]
LP and NLP							
Placebo	20/693 (20/23-HM)	20/34 (20/18-20/321)	20/17 (20/13-57)	7	20/17 (20/16-20/32) [20/12-20/60]	2 (28.6) [8.2-64.1]	2 (28.6) [8.2-64.1]
Intravenous corticosteroid	20/796 (20/16-HM)	20/69 (20/16-HM)	20/35 (20/14-CF)	9	20/35 (20/15-20/502) [20/13-HM]	6 (66.7) [35.4-87.9]	4 (44.4) [18.9-73.3]

Abbreviations: CF, count fingers; HM, hand motion; LP, light perception; NLP, no light perception; VA, visual acuity.

^a^ All computations were performed using logMAR and converted to Snellen VA. The midpoint was used to define breaks between VA categories (eg, 20/65) to account for those participants with a baseline VA that fell between categories (eg, between 20/60 and 20/70).

^b^ Indicates Wilson 95% CI.

## Discussion

We used the ONTT to develop models of visual outcomes in patients with typical ON. We found that baseline VA and CS were the primary variables associated with VA and CS at 1 year, and there was no evidence of treatment benefit at 1 year. Model discrimination was low, because VA and CS outcomes were nearly universally good for all participants (78.1% with 20/20 or better and 94.8% with 20/40 or better). To place these findings into context, a patient with a baseline VA of 20/400 would be expected to read 3 fewer letters on an ETDRS eye chart at 1 year compared with a patient with a baseline VA of 20/40. In most circumstances, this translates into less than a single line of difference on a Snellen eye chart and is unlikely to be a clinically meaningful difference. Although an intravenous corticosteroid treatment benefit was seen at 15 days in participants with a baseline VA better than CF, the effect size was approximately 10 letters on an ETDRS chart, which is of uncertain clinical significance. Furthermore, in participants with baseline VA of CF or worse, we found no evidence of a 15-day treatment effect. Similar results were observed with CS. Collectively, this information about long- and short-term outcomes provides new data to inform personalized shared decision-making conversations.

Our study builds on prior work of predicted long-term visual outcomes and treatment benefit. Beck et al^3^ demonstrated that baseline VA was the best predictor of 6-month VA (*P* < .001) in a least squares regression model of baseline VA, age, and treatment assignment. We found that baseline VA continues to be the best variable associated with VA at 1 year in a model of 11 available baseline clinical variables. Multiple sclerosis status was also associated with VA at 1 year for people with baseline VA of CF or less, but we would caution that this association may be owing to chance given the small sample size and subsequent revision of MS diagnostic criteria. In addition, Kupersmith et al^14^ evaluated the trend in improvement over time as a predictor of visual outcomes. While this information is important, it does not inform the decision to use or not use corticosteroids at baseline. Our study can be used to inform treatment decisions for patients with typical optic neuritis.

To apply our results to estimate the 15-day and 1-year VA in patients at the time of baseline evaluations, clinicians can make the specific calculation using the linear regression formula in Tables 2 and 3 or, more simply, use our Table 4 scenarios. For example, a patient with typical optic neuritis and a VA of 20/70 at presentation, similar to the average ONTT participant, could be counseled that their estimated VA in 2 weeks is approximately 20/25 without corticosteroids and 20/20 if they choose to use corticosteroids. At 1 year, their estimated VA is 20/20 regardless of treatment but may range from 20/20 to about 20/40. In participants with 20/70 to 20/160 VA in the trial, 28 of 36 (77.8%) were 20/20 or better and 34 of 36 (94.4%) were 20/40 or better at 1 year. The information presented in Table 4 could also draw a clinician’s attention to the fact that a patient may not have typical optic neuritis if they are not recovering in the expected manner during the first month.

Because the early benefit of corticosteroids was small when present at all, patients and clinicians should weigh this information against the potential harms of corticosteroids. In the ONTT, approximately 50% of patients experienced at least mild adverse effects, such as insomnia.^15^ Other inconveniences of corticosteroids were not captured, including hospitalizations, travel to infusion centers, or difficulty taking more than 20 tablets per day of prednisone if a high-dose oral regimen was used.^16^ Among patients using short-term, low-dose corticosteroids (median prednisone equivalent dose, 20 mg/d) for other medical reasons, a recent study^17^ demonstrated increased incidence rates of sepsis, venous thromboembolism, and fracture within 30 days. Collectively, these findings should call into question whether corticosteroids should be used at all in patients with typical optic neuritis—a practice that is otherwise reported by 90% of clinicians.^5^

### Limitations

Our study has important limitations. First, the ONTT consisted primarily of women who were white with typical demyelinating optic neuritis. Therefore, our findings cannot be generalized to patients with atypical optic neuritis, including neuromyelitis optica and myelin oligodendrocyte glycoprotein–associated optic neuritis or those who would not have met the ONTT inclusion criteria. We also did not find an association between symptom duration and long-term visual function but cannot address whether hyperacute corticosteroid treatment before the onset of vision loss would have aborted the attack.^18^ Second, the ONTT data are more than 25 years old. Although little evidence suggests that the diagnosis or natural history of optic neuritis has changed substantially over time, external validation in a more modern cohort is needed. Third, few patients with poor visual function both at baseline and at 1 year were included. Further work should be undertaken to understand why some people with poor visual function do not substantially recover and to try to estimate who might be at risk. Third, we did not include optic neuritis recurrences as a variable in our models, because that information is unknown to the clinician at the time of initial presentation when corticosteroid treatment decisions are made. An unanticipated finding of the ONTT was that patients treated with oral corticosteroids were more likely to have an optic neuritis relapse. This finding is potentially important because those with a relapse were more likely to have a poor outcome. Fourth, MS is now diagnosed using more sensitive criteria (2017 McDonald criteria).^19^ Therefore, the association between MS and optic neuritis visual outcomes should be evaluated in a study that can apply the 2017 McDonald criteria. Last, because the ONTT was underpowered to detect serious corticosteroid harms, we cannot use this data set to estimate risks of individual-level harms.

## Conclusions

Shared decision-making is a central part of patient-centered health care and is particularly important when the benefits of an intervention have a marginal effect on outcomes and harms are an important concern. Personalizing the ONTT data to inform clinicians and patients about treatment benefits and long-term outcome has the potential to support both evidence-based care and shared decision-making. Further studies are needed to understand those patients with poor recovery and, importantly, to integrate information about corticosteroid benefits with estimates of corticosteroid harms.

## Notes

Supplement. **eTable.** Predicted High-Contrast Acuity at 15 and 30 Days and 1 Year Based on Presenting VA and Treatment, Observed Tukey Summary of VA at 1 Year, and Count/Percentage Worse Than 20/20 and 20/40 at 1 Year With Wilson Confidence Interval
