# Supplementary material for: Association of Individual-Level Factors With Visual Outcomes in Optic Neuritis: Secondary Analysis of a Randomized Clinical Trial
Source: JAMA Netw Open. 2020 May 7;3(5):e204339. doi: 10.1001/jamanetworkopen.2020.4339 (PMC7206503; doi:10.1001/jamanetworkopen.2020.4339)
Supplement: Supplement. — eTable. Predicted High-Contrast Acuity at 15 and 30 Days and 1 Year Based on Presenting VA and Treatment, Observed Tukey Summary of VA at 1 Year, and Count/Percentage Worse Than 20/20 and 20/40 at 1 Year With Wilson Confidence Interval [file jamanetwopen-3-e204339-s001.pdf]

Supplementary Online Content

De Lott LB, Burke JF, Andrews CA, et al. Association of individual-level factors with visual outcomes in optic neuritis: secondary analysis of a randomized clinical trial. *JAMA Netw Open*. 2020;3(5):e204339. doi:10.1001/jamanetworkopen.2020.4339

eTable. Predicted High-Contrast Acuity at 15 and 30 Days and 1 Year Based on Presenting VA and Treatment, Observed Tukey Summary of VA at 1 Year, and Count/Percentage Worse Than 20/20 and 20/40 at 1 Year With Wilson Confidence Interval

This supplementary material has been provided by the authors to give readers additional information about their work.

©2020 De Lott LB et al. *JAMA Network Open*.

eTable. Predicted High-Contrast Acuity at 15 and 30 Days and 1 Year Based on Presenting VA and Treatment, Observed Tukey Summary of VA at 1 Year, and Count/Percentage Worse Than 20/20 and 20/40 at 1 Year With Wilson Confidence Interval

A. UK Snellen scale

Presenting Visual Acuity	Treatment	Predicted 15 day VA (95% PI)	Predicted 30-day VA (95% PI)	Predicted 1-year VA (95% PI)	Observed 1-year VA in ONTT			
					Tukey Summary: (min, Q1, median, Q3, max)	N	Worse than 6/6 N, % (CI)	Worse than 6/12 N, % (CI)
Better than 6/9	Placebo	6/6 (6/4, 6/20)	6/5 (6/4, 6/9)	6/5 (6/3, 6/7)	6/ (3, 4, 5, 5, 10)	47	7 14.9% (7.4, 27.7)	0 0.0% (0.0, 7.6)
	IV corticosteroid	6/5 (6/4, 6/8)	6/5 (6/3, 6/7)	6/5 (6/3, 6/7)	6/ (3, 4, 5, 5, 7)	35	2 5.7% (1.6, 18.6)	0 0.0% (0.0, 9.9)
6/9 to 6/18	Placebo	6/7 (6/4, 6/38)	6/6 (6/4, 6/13)	6/5 (6/3, 6/9)	6/ (3, 4, 5, 6, 8)	26	6 23.1% (11.0, 42.0)	0 0.0% (0.0, 12.9)
	IV corticosteroid	6/5 (6/4, 6/14)	6/5 (6/3, 6/11)	6/5 (6/3, 6/8)	6/ (3, 4, 5, 6, 104)	29	6 20.7% (9.8, 38.4)	1 3.4% (0.6, 17.2)
6/21 to 6/48	Placebo	6/7 (6/4, 6/79)	6/6 (6/4, 6/20)	6/5 (6/3, 6/12)	6/ (3, 4, 5, 6, 173)	15	4 26.7% (10.9, 51.9)	2 13.3% (3.7, 37.9)
	IV corticosteroid	6/6 (6/4, 6/30)	6/5 (6/3, 6/17)	6/5 (6/3, 6/11)	6/ (3, 4, 5, 5, 10)	21	4 19.0% (7.7, 40.0)	0 0.0% (0.0, 15.5)
6/60 to 6/120	Placebo	6/9 (6/5, 6/175)	6/6 (6/4, 6/33)	6/5 (6/4, 6/17)	6/ (4, 5, 5, 6, 66)	10	2 20.0% (5.7, 51.0)	1 10.0% (1.8, 40.4)
	IV corticosteroid	6/7 (6/4, 6/66)	6/6 (6/4, 6/28)	6/5 (6/3, 6/15)	6/ (3, 4, 5, 5, 20)	14	2 14.3% (4.0, 39.9)	2 14.3% (4.0, 39.9)
6/150 to 6/300	Placebo	6/10 (6/5, 6/354)	6/7 (6/4, 6/51)	6/5 (6/4, 6/22)	6/ (4, 4, 5, 6, 9)	15	3 20.0% (7.0, 45.2)	0 0.0% (0.0, 20.4)
	IV corticosteroid	6/8 (6/4, 6/135)	6/6 (6/4, 6/44)	6/5 (6/3, 6/20)	6/ (3, 4, 5, 5, 27)	17	2 11.8% (3.3, 34.3)	1 5.9% (1.0, 27.0)
CF or HM	Placebo	6/16 (6/7, HM)	6/7 (6/5, 6/204)	6/7 (6/4, 6/12)	6/ (4, 5, 7, 8, 13)	13	7 53.8% (29.1, 76.8)	1 7.7% (1.4, 33.3)
	IV corticosteroid	6/7 (6/5, 6/220)	6/6 (6/4, 6/141)	6/6 (6/5, 6/12)	6/ (5, 6, 6, 7, 12)	12	6 50.0% (25.4, 74.6)	0 0.0% (0.0, 24.2)
LP or NLP	Placebo	6/208 (6/7, HM)	6/10 (6/5, 6/96)	6/5 (6/4, 17)	6/ (3, 5, 5, 10, 18)	7	2 28.6% (8.2, 64.1)	2 28.6% (8.2, 64.1)
	IV corticosteroid	6/239 (6/5, HM)	6/21 (6/5, HM)	6/10 (6/4, CF)	6/ (4, 5, 10, 151, HM)	9	6 66.7% (35.4, 87.9)	4 44.4% (18.9, 73.3)

©2020 De Lott LB et al. *JAMA Network Open*.

B. logMAR SCALE

Presenting Visual Acuity	Treatment	Predicted 15 day VA (95% PI)	Predicted 30-day VA (95% PI)	Predicted 1-year VA (95% PI)	Observed 1-year VA in ONTT			
					Tukey Summary: (min, Q1, median, Q3, max)	N	Worse than 0.0 N, % (CI)	Worse than 0.3 N, % (CI)
Better than 0.18	Placebo	-0.01 (-0.16, 0.52)	-0.06 (-0.20 0.16)	-0.12 (-0.25, 0.08)	(-0.26, -0.18, -0.12, -0.06, 0.20)	47	7 14.9% (7.4, 27.7)	0 0.0% (0.0, 7.6)
	IV corticosteroid	-0.12 (-0.22, 0.10)	-0.10 (-0.26, 0.09)	-0.12 (-0.26, 0.04)	(-0.24, -0.16, -0.12, -0.06, 0.08)	35	2 5.7% (1.6, 18.6)	0 0.0% (0.0, 9.9)
0.18 to 0.48	Placebo	0.04 (-0.15, 0.80)	-0.03 (-0.18, 0.33)	-0.11 (-0.24, 0.19)	(-0.24, -0.14, -0.04, 0.00, 0.14)	26	6 23.1% (11.0, 42.0)	0 0.0% (0.0, 12.9)
	IV corticosteroid	-0.07 (-0.20, 0.38)	-0.08 (-0.25, 0.26)	-0.11 (-0.26, 0.15)	(-0.28, -0.18, -0.08, 0.00, 1.24)	29	6 20.7% (9.8, 38.4)	1 3.4% (0.6, 17.2)
0.54 to 0.9	Placebo	0.10 (-0.14, 1.12)	-0.00 (-0.17, 0.53)	-0.09 (-0.24, 0.31)	(-0.24, -0.16, -0.10, 0.01, 1.46)	15	4 26.7% (10.9, 51.9)	2 13.3% (3.7, 37.9)
	IV corticosteroid	-0.02 (-0.19, 0.70)	-0.04 (-0.23, 0.46)	-0.10 (-0.25, 0.27)	(-0.26, -0.18, -0.10, -0.04, 0.22)	21	4 19.0% (7.7, 40.0)	0 0.0% (0.0, 15.5)
1.0 to 1.3	Placebo	0.16 (-0.12, 1.46)	0.03 (-0.15, 0.74)	-0.08 (-0.23, 0.44)	(-0.16, -0.10, -0.05, 0.00, 1.04)	10	2 20.0% (5.7, 51.0)	1 10.0% (1.8, 40.4)
	IV corticosteroid	0.04 (-0.18, 1.04)	-0.01 (-0.22, 0.67)	-0.09 (-0.24, 0.40)	(-0.26, -0.18, -0.09, -0.06, 0.52)	14	2 14.3% (4.0, 39.9)	2 14.3% (4.0, 39.9)
1.4 to 1.7	Placebo	0.21 (-0.11, 1.77)	0.06 (-0.14, 0.93)	-0.07 (-0.22, 0.56)	(-0.22, -0.16, -0.08, -0.02, 0.18)	15	3 20.0% (7.0, 45.2)	0 0.0% (0.0, 20.4)
	IV corticosteroid	0.10 (-0.16, 1.35)	0.02 (-0.21, 0.86)	-0.07 (-0.24, 0.52)	(-0.24, -0.14, -0.08, -0.04, 0.66)	17	2 11.8% (3.3, 34.3)	1 5.9% (1.0, 27.0)
CF or HM	Placebo	0.42 (0.06, 2.39)	0.09 (-0.11, 1.53)	0.04 (-0.16, 0.29)	(-0.18, -0.06, 0.04, 0.10, 0.34)	13	7 53.8% (29.1, 76.8)	1 7.7% (1.4, 33.3)
	IV corticosteroid	0.08 (=0.12, 1.56)	0.03 (=0.19, 1.37)	0.02 (-0.08, 0.29)	(-0.10, -0.02, 0.02, 0.04, 0.30)	12	6 50.0% (25.4, 74.6)	0 0.0% (0.0, 24.2)
LP or NLP	Placebo	1.54 (0.06, 2.59)	0.23 (-0.04, 1.21)	-0.08 (-0.20, 0.46)	(-0.24, -0.09, -0.08, 0.20, 0.48)	7	2 28.6% (8.2, 64.1)	2 28.6% (8.2, 64.1)
	IV corticosteroid	1.60 (-0.10, 2.30)	0.54 (-0.11, 2.30)	0.24 (-0.16, 1.96)	(-0.18, -0.12, 0.24, 1.40, 2.30)	9	6 66.7% (35.4, 87.9)	4 44.4% (18.9, 73.3)

©2020 De Lott LB et al. *JAMA Network Open*.

C. Glasgow acuity card notation

Presenting Visual Acuity	Treatment	Predicted 15 day VA (95% PI)	Predicted 30-day VA (95% PI)	Predicted 1-year VA (95% PI)	Observed 1-year VA in ONTT			
					Tukey Summary: (min, Q1, median, Q3, max)	N	Worse than 1.0 N, % (CI)	Worse than 0.7 N, % (CI)
Better than 0.82	Placebo	1.01 (0.48, 1.16)	1.06 (0.84, 1.20)	1.12 (0.92, 1.25)	(0.80, 1.06, 1.12, 1.18, 1.26)	47	7 14.9% (7.4, 27.7)	0 0.0% (0.0, 7.6)
	IV corticosteroid	1.12 (0.90, 1.22)	1.10 (0.91, 1.26)	1.12 (0.96, 1.26)	(0.92, 1.06, 1.12, 1.16, 1.24)	35	2 5.7% (1.6, 18.6)	0 0.0% (0.0, 9.9)
0.82 to 0.52	Placebo	0.96 (0.20, 1.15)	1.03 (0.67, 1.18)	1.11 (0.81, 1.24)	(0.86, 1.00, 1.04, 1.14, 1.24)	26	6 23.1% (11.0, 42.0)	0 0.0% (0.0, 12.9)
	IV corticosteroid	1.07 (0.62, 1.20)	1.08 (0.74, 1.25)	1.11 (0.85, 1.26)	(-0.24, 1.00, 1.08, 1.18, 1.28)	29	6 20.7% (9.8, 38.4)	1 3.4% (0.6, 17.2)
0.46 to 0.1	Placebo	0.90 (-0.12, 1.14)	1.00 (0.47, 1.17)	1.09 (0.69, 1.24)	(-0.46, 0.99, 1.10, 1.16, 1.24)	15	4 26.7% (10.9, 51.9)	2 13.3% (3.7, 37.9)
	IV corticosteroid	1.02 (0.30, 1.19)	1.04 (0.54, 1.23)	1.10 (0.73, 1.25)	(0.78, 1.04, 1.10, 1.18, 1.26)	21	4 19.0% (7.7, 40.0)	0 0.0% (0.0, 15.5)
0.0 to -0.3	Placebo	0.84 (-0.46, 1.12)	0.97 (0.26, 1.15)	1.08 (0.56, 1.23)	(-0.04, 1.00, 1.05, 1.10, 1.16)	10	2 20.0% (5.7, 51.0)	1 10.0% (1.8, 40.4)
	IV corticosteroid	0.96 (-0.04, 1.18)	1.01 (0.33, 1.22)	1.09 (0.60, 1.24)	(0.48, 1.06, 1.09, 1.18, 1.26)	14	2 14.3% (4.0, 39.9)	2 14.3% (4.0, 39.9)
-0.4 to -0.7	Placebo	0.79 (-0.77, 1.11)	0.94 (0.07, 1.14)	1.07 (0.44, 1.22)	(0.82, 1.02, 1.08, 1.16, 1.22)	15	3 20.0% (7.0, 45.2)	0 0.0% (0.0, 20.4)
	IV corticosteroid	0.90 (-0.35, 1.16)	0.98 (0.14, 1.21)	1.07 (0.48, 1.24)	(0.34, 1.04, 1.08, 1.14, 1.24)	17	2 11.8% (3.3, 34.3)	1 5.9% (1.0, 27.0)
CF or HM	Placebo	0.58 (-1.39, 0.94)	0.91 (-0.53, 1.11)	0.96 (0.71, 1.16)	(0.66, 0.90, 0.96, 1.06, 1.18)	13	7 53.8% (29.1, 76.8)	1 7.7% (1.4, 33.3)
	IV corticosteroid	0.92 (-0.56, 1.12)	0.97 (-0.37, 1.19)	0.98 (0.71, 1.08)	(0.70, 0.96, 0.98, 1.02, 1.10)	12	6 50.0% (25.4, 74.6)	0 0.0% (0.0, 24.2)
LP or NLP	Placebo	-0.540 (-1.592, 0.938)	0.770 (-0.205, 1.040)	(1.080 (0.544, 1.198)	(0.52, 0.80, 1.08, 1.09, 1.24)	7	2 28.6% (8.2, 64.1)	2 28.6% (8.2, 64.1)
	IV corticosteroid	-0.600 (-1.300, 1.096)	0.460 (-1.300, 1.109)	0.760 (-0.964, 1.164)	(-1.30, -0.40, 0.76, 1.12, 1.18)	9	6 66.7% (35.4, 87.9)	4 44.4% (18.9, 73.3)

©2020 De Lott LB et al. *JAMA Network Open*.
